# Deep Quality Assessment of a Solar Reflector Based on Synthetic Data: Detecting Surficial Defects from Manufacturing and Use Phase

**DOI:** 10.3390/s20195481

**Published:** 2020-09-24

**Authors:** Alexios Papacharalampopoulos, Konstantinos Tzimanis, Kyriakos Sabatakakis, Panagiotis Stavropoulos

**Affiliations:** Laboratory for Manufacturing Systems and Automation, Department of Mechanical Engineering and Aeronautics, University of Patras, 265 04 Patras, Greece; apapacharal@lms.mech.upatras.gr (A.P.); tzimanis@lms.mech.upatras.gr (K.T.); sabatakakis@lms.mech.upatras.gr (K.S.)

**Keywords:** defect detection, vision techniques, image recognition, neural networks, parabolic reflector, surface monitoring

## Abstract

Vision technologies are used in both industrial and smart city applications in order to provide advanced value products due to embedded self-monitoring and assessment services. In addition, for the full utilization of the obtained data, deep learning is now suggested for use. To this end, the current work presents the implementation of image recognition techniques alongside the original the quality assessment of a Parabolic Trough Collector (PTC) reflector surface to locate and identify surface irregularities by classifying images as either acceptable or non-acceptable. The method consists of a three-step solution that promotes an affordable implementation in a relatively small time period. More specifically, a 3D Computer Aided Design (CAD) of the PTC was used for the pre-training of neural networks, while an aluminum reflector surface was used to verify algorithm performance. The results are promising, as this method proved applicable in cases where the actual part was manufactured in small batches or under the concept of customized manufacturing. Consequently, the algorithm is capable of being trained with a limited number of data.

## 1. Introduction

In the modern world, the industrial sector makes a significant effort to reduce the environmental impact that arises from the excessive consumption of fossil fuels to provide the required energy to run production [1]. Small industries and individuals are trying to take advantage of the renewable energy sources while large scale industries face difficulties to integrate such systems due to the enormous investment required to transform one of the solar, wind, hydro, tidal, geothermal, and biomass energy into electrical energy or other types of useful energy. Some of the abovementioned energy sources can be transformed to electricity while others remain as is, taking advantage of the heat transfer mechanisms in order to heat up or cool down surfaces or working fluids.

Meanwhile, modern techniques are employed to evaluate the working condition of the collector and to ensure the performance operation of the solar tracking device. The zero-defect manufacturing (ZDM) approach is related to the reduction of the defective products developed in a production line while there is also a similar approach for the life cycle of the product in order to prevent inefficient conditions due to failures [2]. Several techniques are used towards this direction; real-time monitoring, development of digital models that work with inputs directly from the machine, and tools for the optimization of processes and advanced quality assessment techniques are some of the technological achievements of the present that are applied to minimize the faulty production and ensure financial and environmental sustainability. Currently, machine vision techniques and machine learning technologies are used in the industrial sector in order to investigate the working conditions of equipment by analyzing data obtained directly from the machine Programmable Logic Controller (PLC) or by analyzing images. Machine vision and image recognition techniques are mainly used for safety reasons as well as to locate irregularities or cracks at the production or working phase of a product [3]. The machine vision process begins with the installation of a high-resolution camera at a stiff and fixed position ensuring the stability of the camera mount. The camera scans the desired surface while transmitting the obtained images to a computer for further analysis. Image recognition algorithms are responsible to process the images and categorize them based on their characteristics. Neural networks are then employed to learn how to distinguish the faulty and acceptable products [4].

This study aims to present an inexpensive, fast, and accurate way of pre-training an image recognition algorithm for the defect detection on the reflector surface of a parabolic trough collector with the aid of 3D cad and a real scaled final product. It is noted though that the “scaled” character of the prototype implies the use of a particular portion of the reflector, which has specific dimensions proportional to the field of view of the images in the dataset. Various kinds of defects have been considered that are usually generated either during manufacturing or during use phase. Moreover, the goal of this approach is to detect the defects no matter where they come from in the acquired segments of the panel. Further, a second stage could be added to evaluate the aggregated impact from all the defects on the performance, but this exceeds the purposes of the current document. Neural networks are employed for the classification of images into acceptable and non-acceptable (OK/NOK).

The obtained results are promising, as this method proved to be applicable in cases where the actual part was manufactured at small batches or under the concept of customized manufacturing and, consequently, the algorithm was capable of being trained with a limited number of data. In the following section, the application of machine vision techniques in the industrial sector can be found, proving the usefulness of convolutional neural networks (CNNs) in applications. In Section 3, the proposed approach and methodology are presented. Therein, the experimental procedure that we followed to evaluate the results of the image recognition algorithm, as well as the algorithm core itself, are mentioned. The results and the conclusions can be found in Section 4 and Section 5, respectively.

## 2. State of the Art

Machine vision techniques and their significance in the industrial sector are presented in detail in [5]. The improved productivity, the reliable quality assessment, the high performance either of equipment or for the product, online integration, and the non-contact measurements are considered as important features in vision systems. Out of line measurements reduce productivity rates and are employed only when the production process has finished [6]. Industries are trying to avoid this type of measurements because it is a passive method, which involves a lot of material waste without being financial and environmentally sustainable. Moreover, welding industries [4], food industries [7], and micro-electronic industries [8] benefit from vision systems, succeeding in providing high quality products and services by reducing their environmental footprint and faulty products. Additional works have examined the integration of vision systems in the industrial world. Zhang et al. [9] presented the design and testing procedure of an automatic inspection system for the defect detection on metal’s surface, while Giuseppe et al. [10] described an online image-based inspection system for the inspection of electromechanical parts.

In addition, Ming Chang et al. [11] presented the development of an optical inspection platform that combines parallel image processing with high resolution opto-mechanical module for the detection of defects of touch panel glass. This work proposes the implementation of a custom illumination source to improve the scattering efficiency of the surface, while improving the contrast of the obtained images and the performance of the vision inspection system. Finally, Çelik et al. [12] provides a machine vision system to achieve fabric inspection and defect classification processes automatically. The developed vision system consists of an image acquisition hardware and an image processing software for real-time defect detection and classification of algorithms with great accuracy.

Vision systems are applied to for the inline or out of line image acquisition. These images are classified into several categories based on their special characteristics [13]. According to [14], neural networks can be considered as a mean of supervised learning. Networks are used to make a prediction of output values based on the input values for a known system.

The present work aims to experimentally investigate the application of vision systems on the aluminum reflector surface of a parabolic trough collector installed on the roof of a small industry. In this particular work, the solar collector is used to heat up heat transfer fluids, namely hot water for domestic use or working fluids for industrial use. Machine vision technologies are applied to detect irregularities with the aid of image recognition algorithms while taking advantage of deep learning methods such as neural networks. The significance of this work identified the automatic recognition of surface failures which, up to this moment, affect the performance of solar collector making them an inefficient method of transforming the solar energy to useful energy with high initial cost. Moreover, this particular methodology has been proven to be highly efficient, taking into account the reduction of the experiments needed to train a CNN, which is a computationally demanding method proven to be highly efficient [15,16], as it is able to choose the features out of the images. In addition, the cost of the overall system (taking into account high information content sensors, such as cameras, the current micro-controllers, and training experiments) is proven to be much smaller than any other similar approach. The following section includes the details of the development of this vision system, as well as the experimental work used for the validation of the image recognition algorithm.

## 3. Approach

### 3.1. Quality Control System Design Approach Based On 5C Architecture

**Figure 1:**
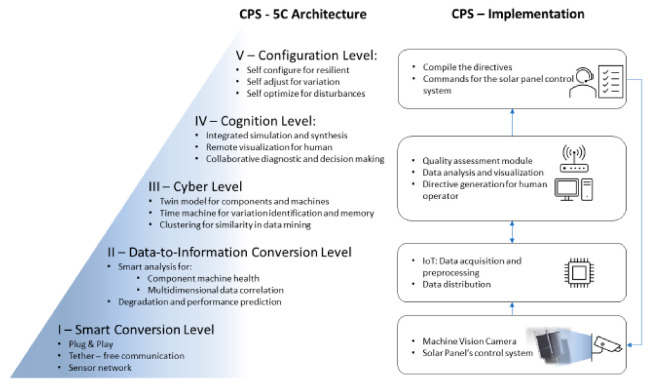
5C architecture [17].

In the current study, the authors were based on 5C architecture, as described in Figure 1, which envisioned an online non-invasive automated quality control system able to identify defective surfaces of solar panel reflectors [16].

As such, image processing techniques and neural networks, accompanied by all the means described in the 5C approach, are employed to characterize the quality of the reflector surface of a parabolic trough collector [18]. As a first step for the proposed solution, a 3D Computer Aided Design (CAD) was used in order to provide the necessary images for their classification into OK and NOK by neural networks. This technique consists of a low-cost solution that saves time from the training phase while improving the range of defects that can be used for neural network training. Then, for algorithm validation, a 3D printed parabolic collector was created in a scale of the original part with possible defects designed on it.

As depicted in Figure 1, the smart connection level includes a single sensor in the form of a 20 MP, 18 fps Complementary Metal Oxide Semiconductor (CMOS) machine vision colored camera (FLIR Systems, Wilsonville, Oregon, United States), which targets the reflector’s surface from various angles, depending on the physical setup. The image capturing process is handled by an Internet of Things (IoT) module (NVIDIA Jetson TX2, Nvidia Corporation, United States, California, Santa Clara) connected to the camera via a USB3.0. The IoT is used in order to generalize the technique so that it is able to be applied in the case where there are multiple collectors installed. This was cleared out in the approach. Additionally, a custom application, developed with the FLIR’s Spinnaker Software Development Kit SDK (FLIR Systems, Wilsonville, Oregon, United States) and Python, enables image acquisition and preprocessing tasks, which are integrated via a data-to-information conversion level in the system. Following this, the cognition level, as the name implies, is equipped with the quality assessment module, which identifies defective solar panels (OK/NOK) based on a machine learning model that utilizes as inputs the data emerged from previous stages. Herein, the output of the model along with local weather-oriented data fetched from the web were visualized and analyzed. Directives were generated for aiding the human operators. At the configuration level, the control loop is closed with the human operators applying the appropriate controls to the system. All of the above resulted in a module that can be fabricated and assembled in parallel to the actual parabolic system and setup seamlessly at the same phase as the reflector.

### 3.2. Acceleration of the Quality Assessment Algorithm’s Development

**Figure 2:**
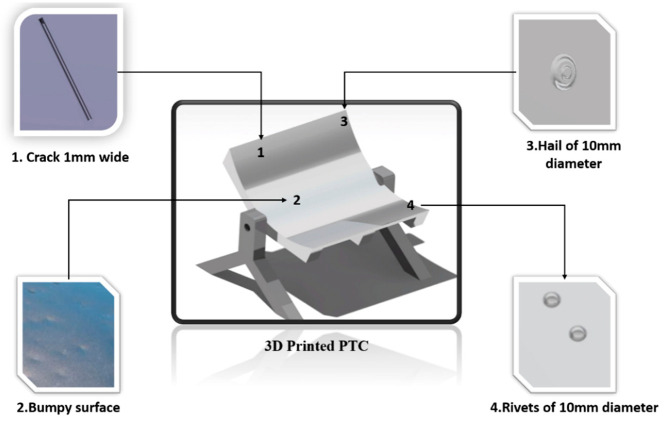
Failures at the Parabolic Trough Collector (PTC) surface.

The main goal of this study was the development of a quality assessment algorithm by utilizing images of a defective solar pane. Thus, the approach taken included the development of a software module able to identify various defects. Considering the data availability at the initial absence of a monitoring system and requirements for its deployment and reliable operation at a low cost, we took advantage of CAD design tools (CATIA) in order to generate a set of realistic images (Figure 2) of defective and non-defective solar panel reflector surfaces based on real ones. This approach enabled the generation of image data at an early stage, which aided the process of designing and implementing an image classification algorithm from an early stage, ready to be deployed at moment’s notice. This approach is foreseen to enable the development of an image classification algorithm with highly reduced real data.

The dataset built had an objective to recreate uncertainty present in real cases. This uncertainty included variations in the positioning of the defect on the reflector, the size and the shape of the defect, the field of view of the camera with respect to the defect and reflector, and the coexistence with other defects and fundamental self-shading. It was logical, then, that the predicting capability of the system was restricted due to a non-fully representative training (and evaluation) dataset; however, these were proven to be adequate for the current work.

By adopting the concept of transferred learning [19], the first generation of the quality assessment model (trained with CAD images) was retrained with a smaller size dataset, emerged from photos of downscaled prototypes of defective/non defective solar panels. In this way, the initial model’s cognition capabilities were boosted and the learning process’ efficiency was accelerated and optimized. In the last stage of this hybrid development process—which was parallel to installing the monitoring system and collecting a tiny amount of real images of defective panels—a final version of the model was developed.

### 3.3. Case Study & Experimental Setup

A reflector is the key component of solar tracking mechanisms in applications where solar irradiation is concentrated on a determined focal point. If the surface is scratched or bumpy then the solar energy is not reflected at the desired point, wasting energy and making the mechanism inefficient. The proposed tools tend to replace the conventional visual inspection with machine vision technologies. At the same time, the life cycle monitors the reflector surface with the aid of image recognition techniques, which presents significant benefits. As described above, image recognition algorithms divide images into smaller figures and the trained neural networks identify the defect much faster and with higher accuracy.

**Figure 3:**
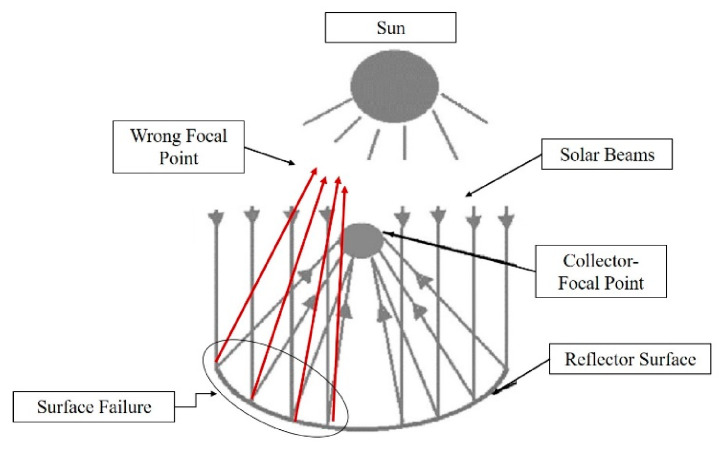
PTC reflector light beams.

The reflector surface was designed in order to provide 1500 W at the focal point. The focal point was 870 mm above the support structure of the parabolic trough collector. The surface covered from the PTC was 12 m^2^. It was financially and environmentally inefficient to create such a surface for the training of the neural network. To this end, a 3D printed Parabolic Trough Collector (PTC) was developed in scale with a number of defects on it and was mounted with features such as rivets (Figure 2). Each one of the defects can lead to a decrease of average reflectivity because of the different directions that solar beams follow, as can be seen in Figure 3.

**Figure 4:**
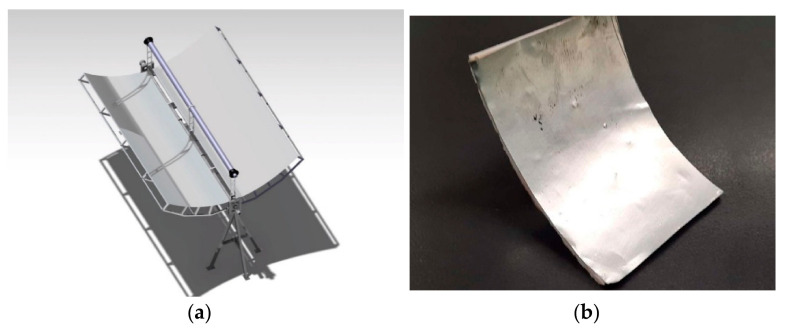
(**a**) 3D CAD for pre-training; (**b**) product for validation.

The parabolic surface was covered with an aluminum reflective surface in order to imitate the real surface properties and assist the algorithm validation process. An aluminum sheet was utilized for this purpose (Figure 4b). For the real structure, a Digital Single-Lens Reflex (DSLR) camera was mounted above the PTC structure, scanning the collector surface. The pre-training of the NN was achieved with the aid of renders from the 3D CAD. Figure 4a depicts the 3D CAD of the PTC. The scaled structure was printed for the pre-training phase and for algorithm validation.

### 3.4. Model Selection

The quality assessment of solar panels fell into the image classification category [20], a task which was accompanied by a number of generic and application-specific challenges. The high dimensional nature of the image data introduced difficulties during the development (training) of machine learning and statistical models, demanding in most cases the utilization of feature extraction/selection methods to avoid overfitting and limit computational costs [19,21]. Beyond these, challenges also originated from intra-class, scale, and viewpoint variations of object of interest in an image, which in the context of this application translated to different defect types found in solar panels, as well as various parameters utilized by the image acquisition system (e.g., viewing angle/distance, optics etc.). Other factors such as occlusion, illumination, and background clutter were tested for robustness in most image classification tasks. Thus, they were expected in the current application as images of solar panels, as they were included in an excessive amount of reflection and had high contrast.

A series of machine learning algorithms [22], such as decision trees, SVM, and NN have been successfully used for performing image classification tasks. However, they typically require a priori, extensive preprocessing in order to normalize and reduce the dimensions of the input data [23]. These tasks are time-consuming and include the development of heuristic application-specific algorithms [24]. In addition, the absence of mechanisms in the spatial coherence of image data makes these approach unsuitable for this application [25].

According to the aforementioned points, we concluded that a model based on the convolutional neural networks was most suitable for developing the quality assessment module, as its combined feature extraction and classification into a single trainable model with a manageable number of tunable parameters [26,27,28]. Ensuring a substantial amount of data, the model was expected to be invulnerable regarding its classification capabilities due to the input’s data variations. In addition, the parametric nature of the model [29] meant that the concept of transferred learning could be applied according to the envisioned training process [30,31].

### 3.5. Model Development Utilizing a Synthetic Dataset

**Figure 5:**
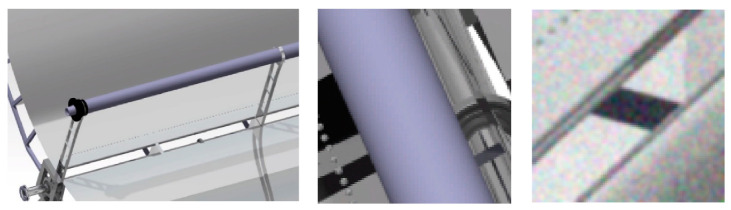
Initial CAD render (**left**), image segment (**center**), and augmented image segment (**right**).

For the training and cross validation of the initial model (M1.1), a set of 110 CAD renders of solar panels (50% artificially defective) were considered. Because these images had high resolution, the resulting dataset was configured by a few numbers of measurements (i.e., number of CAD images) of high dimensional data vectors. Generally, according to the literature [32], training examples were fewer compared to their dimensions, and the CNN training process led to an overfitted model. In addition, the recognition capabilities of such a model was degraded in cases when the subject of interest (defect) had an extremely small size compared to the entire input image [33]. Thus, the authors segmented each one of the CAD images into smaller ones to increase the number of data vectors, reduce their dimensions, and make the size of the defects comparable to image size. As a result, a set of 7870 Red-Green-Blue (RGB) images emerged, each of them labeled as OK/NOK based on their content (including or not a single or more defects). One step towards the foreseen enhancement of the model’s invariability was made by performing image augmentation, i.e., by means of color noise and blur addition in a random manner. This step was followed by the partition of 15,740 RGB images into training (70%), validation (21%), and test (9%) sets, as well as by their grayscale conversion, in order to reduce the dimensions of the data. These data were comprised of the synthetic (SC) dataset (Figure 5).

**Figure 6:**
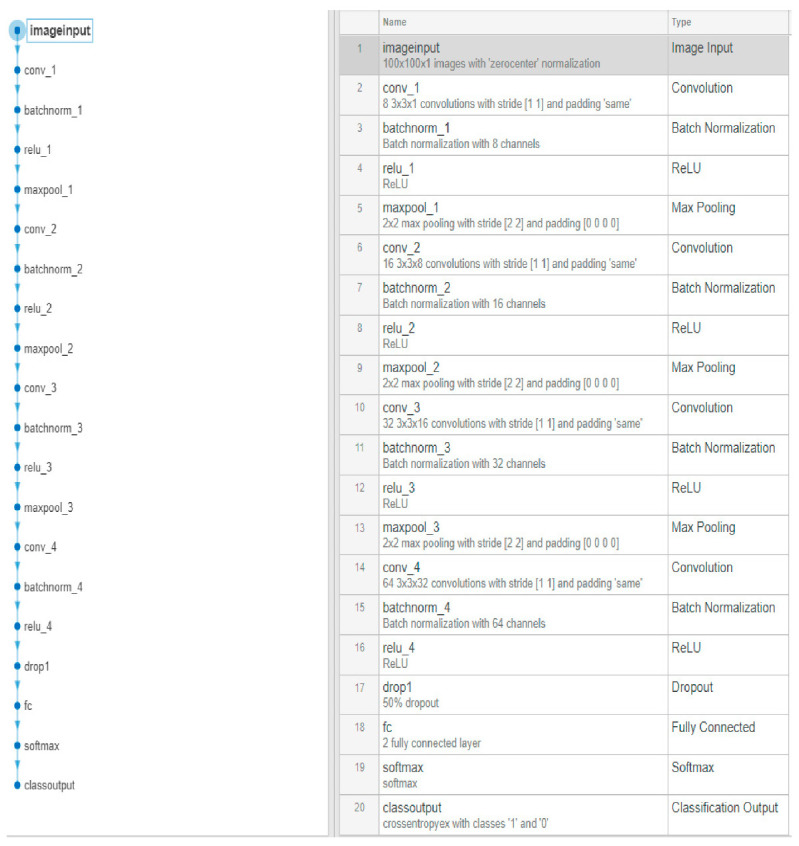
M1.1 architecture.

Regarding the model’s development, a custom convolutional network 20 layers deep was created, as depicted in Figure 6. In total, four convolutional layers with a gradually high number of same-sized filters were included, each followed by a batch normalization layer, an activation function (ReLU), and a max pooling layer [34] for down sampling. A dropout layer [35] was placed right before the series of the fully connected layers. As a stochastic regularization technique, it randomly set 50% of the parameters in the fully connected layer to 0, which prevented the rest from excessively co-adapting to the dataset, which was the result of an overfitting model [36].

A series of training attempts were performed in order to gain performance by utilizing the stochastic gradient descent with momentum (SGDM) [37] as an optimization algorithm of the cross-entropy loss function [38]. The choice of this method-criterion combination was primarily considered since the method was spread in such applications. Thus, the cross-entropy appeared logical for binary classification without excluding other choices. Nevertheless, it was proven to be an efficient combination. By tweaking a number of training options, such as the learning rate, the number of epochs, and the validation frequency, the efficiency of the model’s training process was improved along with its final training and validation performance (Figure 6).

### 3.6. Transfer Learning Using the Trained CNN

**Figure 7:**
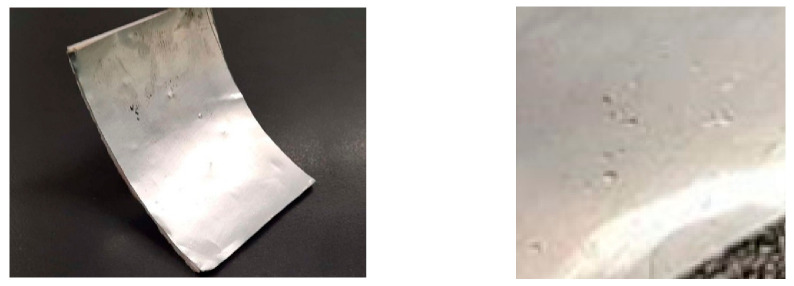
Downscaled solar panel prototype (left); defects close-up (**right**).

A new set of images (i.e., the prototype dataset (PRO)) were segmented from photos that included downscaled solar panel prototypes (Figure 7). These images were utilized in order to apply the concept of transfer learning [29] on the already trained M1.1 model. More specifically, an extra convolution layer was added in order to compensate for the additional data variability inserted from the introduction of real defects, while the last three layers of the M1.1 model were replaced with untrained ones (i.e., fully connected, SoftMax, and classification output). We took advantage of the feature extraction mechanisms already developed, enhancing them by a minor factor and retraining the part of the model related with the classification of the new features that emerged from the additional convolution layer.

## 4. Results

**Figure 8:**
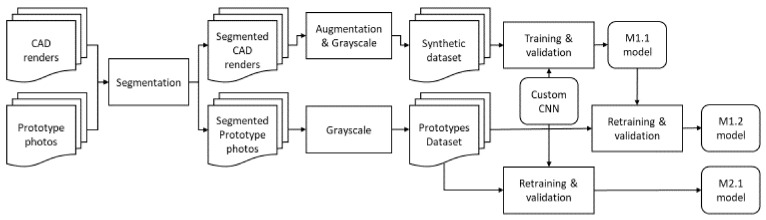
The quality assessment algorithm’s development approach.

In order to evaluate the aforementioned training approach (Figure 8), the M1.2 model’s performance metrics were compared with others as they emerged from the utilization of the SC dataset with the M1.1 model and another model (same architecture) developed with the synthetic data set only, i.e., M2.1. In the following table (Table 1), a series of performance metrics from each model on the available datasets are presented.

As easily concluded from the model’s metrics in the table above (Table 1), M1.2 had a significant performance advantage over M2.1, validating the approach taken for the development of the classification model. The filters were configured during the training of the M1.1 and offered a mechanism for the extraction of high level features, which were further enhanced after adding a new convolutional layer. Thus, the M1.2 model was able to compensate for the differentiation of the defect’s appearance in the PRO dataset. The necessity of this addition was confirmed from the fact that the feature extraction mechanism of M1.1 was not able to successfully recognize these defects, as can be seen from its performance compared to the PRO dataset.

**Figure 9:**
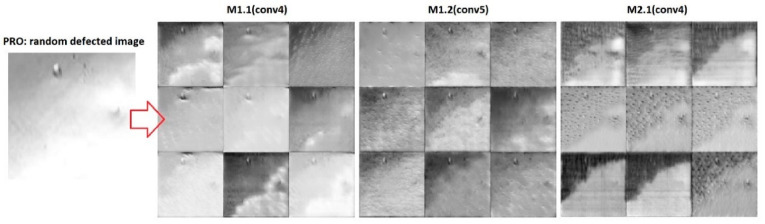
Feature visualization using DeepDream [39].

To further investigate the feature extraction mechanism configured during model training, we made use of the DeepDream [39] to generate a set of images that corresponded to a number of feature maps. Due to strong activation, features were learned by each layer and were highlighted more clearly. Because the M1.1 and M1.2 shared the same feature extraction mechanisms up until the fourth convolutional layer, a set of images was generated (Figure 9) for the fourth, fifth, and fourth convolutional layers of the M1.1, M1.2, and M2.1 models, respectively. Of course, the number of filters was significantly high in these layers, and the first 9 of them were selected. Moreover, images were generated using a randomly selected defected image from the PRO test partition.

Although the M1.1 was trained only for SC data, it was still able to highlight the defect(s) more clearly than M2.1, which learned the features for the complex background in many image close-ups of the downscaled prototypes (i.e., PRO dataset). This may have been an indicator of the overfitting that occurred in M2.1 due to the small training dataset, thus highlighting the significance of this approach for the configuration of a more stable feature extraction mechanism through the transfer learning process, as depicted in M1.2 (conv5).

Nevertheless, as expected, a performance drop was foreseen during the transition from M1.1 to the M1.2 due to factors such as the background of PRO images under specific light conditions appearing as defective surfaces. Further, the shape and random spatial distribution of the defects was in contrast to the symmetry and smoothness described in the SC dataset.

Regarding the performance of the M1.1, we concluded that misclassifications appeared in cases where the defect was located on the panel’s purely illuminated surfaces (e.g., overlapping shadows). In addition, circular shaped components of the solar panels, such as rivets, were also misclassified as defects. The close-up view of image segments deprived the network of spatial information related to their location.

Among the metrics, the specificity of all models was settled at relatively low levels. This fact can be justified by the unbalanced nature of the datasets (i.e., high number of non-defective surfaces) as a false negative (defect existence) prediction influenced by the specificity in contrast to sensitivity.

**Figure 10:**
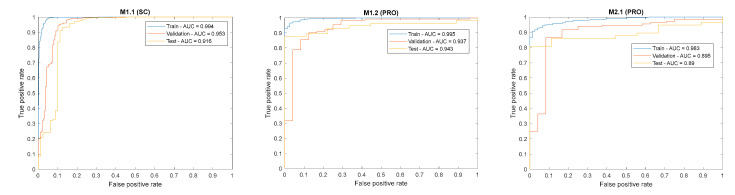
Receiver Operating Characteristic (ROC) curves of the developed models.

The ROC curves and configured AUC qualitatively indicated the class separation capacity for each model (Figure 10) in the case of a balanced dataset.

**Figure 11:**
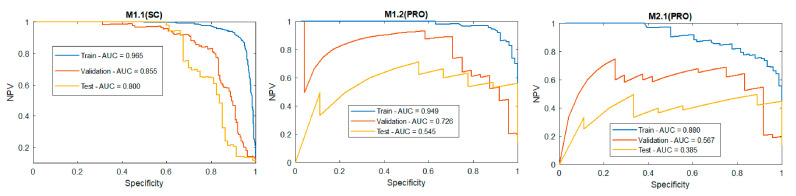
Precision-recall curves.

As can be seen, the M1.2 had a significantly higher chance to predict the right class with an AUC = 0.943 on the test partition. On the other hand, the M2.1’s AUC = 0.89 introduced a higher random factor during classification. However, the highly imbalanced datasets made the ROC curve a deceptive metric with regard to the interpretation of specificity. Precision-recall curves were more indicative for the performance of binary classifiers trained with imbalanced datasets [40,41]. In this case, the class of interest were negative ones (defects), negative predictive rates (NPV), and the specificity curve, all of which were incorporated to graphically investigate the defect detection performance of the models, as depicted in Figure 11.

In general, the performance, excluding the M1.1, was mediocre for detecting the true negative values. This confirms the necessity of a more balanced data set. However, even in this case, the concept of transfer learning (M1.2) provided a significant advantage over the typical training approach.

**Figure 12:**
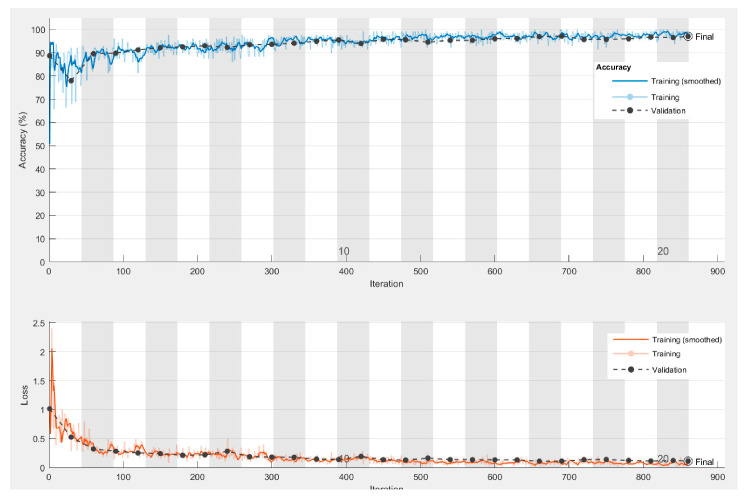
The M1.1 training progress.

As per the network’s architecture, the grayscale conversion of the images built up towards the limitation of the model’s trainable parameters, which avoided overfitting, as can be seen in the training progress figure (Figure 12). However, color-related information was expected to have an impact in the model’s capacity to distinguish defects from other similarly shaped components in the panels and background.

## 5. Conclusions and Future Outlooks

In this study, we implemented modern technology to detect defaults. We aimed to simplify complex maintenance processes that, until now, were executed with human vision techniques, while characterizing the working conditions of the examined equipment was based on human knowledge and experience. Therefore, this study provides a guideline for implementing image recognition techniques to identify surface failures on a solar parabolic trough collector, as well as classifies images into OK and NOK, taking advantage of neural networks and their features. Moreover, the proposed solution was divided in 3 steps, which improved the low-cost integration and reduced training process time. The NN pre-training, with the aid of 3D CAD, was followed by the validation phase, where the developed image recognition algorithm was tested on a 3D printed PTC that represents the 3D CAD in scale. The irregularities on the PTC surface form a real case study scenario was the last step. The development of image recognition algorithms and the training and pre-training phases of neural networks took place on MATLAB. The results showed that it was advantageous to undertake the pre-training process via a 3D CAD, as there was only a minor drop of performance during the validation process for small-scale collectors. Factors such as the reflectivity and shadows on images versus images from CAD appeared to disturb the recognition process. However, the presented approach presented encouraging results and it was worth studying different case studies. Moreover, the use of photorealism in the synthetic data is expected to increase its value in the near future. More specifically, various scenarios of texture and/or shading, as well as specularity, should be used. Furthermore, the concurrent use of other types of defects, such as dirt, needs to be included. Lastly, based on manufacturing, use phase data, and knowledge base, the list of potential defect shapes can be enriched to a further extent.

## Figures and Tables

**Table 1:** Model metrics proving the value of synthetic datasets.

Model	M1.1 (Trained with the Synthetic Dataset)				M1.2 (Extra Convolutional Layer, Retrained with Prototypes)			M2.1 (Initial Architecture, Trained with Prototypes)		
**Dataset**	**SC**			PRO	PRO			PRO		
Partition	Train	Validation	Test	Complete	Train	Validation	Test	Train	Validation	Test
Metrics										
Accuracy	0.978	0.958	0.954	0.806	0.975	0.942	0.909	0.938	0.859	0.848
Sensitivity	0.995	0.987	0.992	0.884	0.993	0.984	0.964	0.984	0.962	0.947
Specificity	0.843	0.725	0.662	0.345	0.868	0.708	0.555	0.671	0.291	0.222
Precision	0.98	0.966	0.958	0.888	0.977	0.948	0.932	0.945	0.881	0.885
F-Measure	0.987	0.976	0.974	0.886	0.985	0.966	0.948	0.964	0.92	0.9153
Geometric Mean	0.916	0.846	0.810	0.552	0.928	0.835	0.732	0.812	0.5297	0.458
